# *HOPX* is a tumor-suppressive biomarker that corresponds to T cell infiltration in skin cutaneous melanoma

**DOI:** 10.1186/s12935-023-02962-2

**Published:** 2023-06-21

**Authors:** Song He, Yu Ding, Zhonghao Ji, Bao Yuan, Jian Chen, Wenzhi Ren

**Affiliations:** 1 Department of Laboratory Animals, College of Animal Sciences, Jilin University, Changchun, 130062 Jilin P.R. China; 2 Department of Basic Medicine, Changzhi Medical College, Changzhi, 046000 Shanxi P.R. China

**Keywords:** *HOPX*, Skin cutaneous melanoma (SKCM), Survival & prognosis, T cell, Drug resistance

## Abstract

**Background:**

Skin cutaneous melanoma (SKCM) is the most threatening type of skin cancer. Approximately 55,000 people lose their lives every year due to SKCM, illustrating that it seriously threatens human life and health. Homeodomain-only protein homeobox (HOPX) is the smallest member of the homeodomain family and is widely expressed in a variety of tissues. HOPX is involved in regulating the homeostasis of hematopoietic stem cells and is closely related to the development of tumors such as breast cancer, nasopharyngeal carcinoma, and head and neck squamous cell carcinoma. However, its function in SKCM is unclear, and further studies are needed.

**Methods:**

We used the R language to construct ROC (Receiver-Operating Characteristic) curves, KM (Kaplan‒Meier) curves and nomograms based on databases such as the TCGA and GEO to analyze the diagnostic and prognostic value of HOPX in SKCM patients. Enrichment analysis, immune scoring, GSVA (Gene Set Variation Analysis), and single-cell sequencing were used to verify the association between HOPX expression and immune infiltration. In vitro experiments were performed using A375 cells for phenotypic validation. Transcriptome sequencing was performed to further analyze HOPX gene-related genes and their signaling pathways.

**Results:**

Compared to normal cells, SKCM cells had low HOPX expression (*p* < 0.001). Patients with high HOPX expression had a better prognosis (*p* < 0.01), and the marker had good diagnostic efficacy (AUC = 0.744). GO/KEGG (Gene Ontology/ Kyoto Encyclopedia of Genes and Genomes) analysis, GSVA and single-cell sequencing analysis showed that HOPX expression is associated with immune processes and high enrichment of T cells and could serve as an immune checkpoint in SKCM. Furthermore, cellular assays verified that HOPX inhibits the proliferation, migration and invasion of A375 cells and promotes apoptosis and S-phase arrest. Interestingly, tumor drug sensitivity analysis revealed that HOPX also plays an important role in reducing clinical drug resistance.

**Conclusion:**

These findings suggest that HOPX is a blocker of SKCM progression that inhibits the proliferation of SKCM cells and promotes apoptosis. Furthermore, it may be a new diagnostic and prognostic indicator and a novel target for immunotherapy in SKCM patients.

**Supplementary Information:**

The online version contains supplementary material available at 10.1186/s12935-023-02962-2.

## Introduction

Melanoma, which results from the deterioration of melanocytes, accounts for approximately 5% of all skin cancers and more than 75% of skin cancer-related deaths. The 5-year relative survival rates for patients with localized or regional disease are 98% and 64%, respectively[1]. The disease is prone to metastasis, and the 5-year survival rate for patients with metastatic melanoma is reduced to 23%[2, 3]. Its frequency is closely related to patient race and geographic area[4–6]. When deteriorated melanocytes are located in the basal layer of the skin surface, skin cutaneous melanoma (SKCM) is formed; otherwise, nonmelanoma skin cancer (NMSC) is formed[7]. NMSC can originate from malignantly transformed melanocytes in the uvea, gastrointestinal tract, urinary tract, and meninges[8–11]. It has been shown that advanced melanoma (stage IV) is highly metastatic and insensitive to therapies such as chemotherapy radiotherapy[12]. Immunotherapy has developed significantly over the past decade and has become an indispensable approach to the treatment of advanced solid tumors[13]. Although advances in immunotherapy have led to improvements in overall and progression-free survival and quality of life in patients with advanced melanoma, there are still some patients with poor treatment outcomes that may be difficult to treat[14–16]. Therefore, it is urgent to search for more specific molecular markers and develop precision medicines to improve the treatment outcomes of SKCM.

As the protein with the smallest homology domain, HOPX is located on human chromosome 4 and consists of seven exons, of which only exons 1, 5, 6, and 7 are fully or partially involved in the transcription of five mRNA splice variants[17, 18]. Its encoded protein is widely expressed in human tissues, with high expression levels in dormant colon stem cells and follicular cells of hair follicles and low expression in lung, breast, and head and neck cancers[19–21]. It has been shown that HOPX is involved in regulating the homeostasis of hematopoietic stem cells[22] and is closely related to tumor proliferation and apoptosis[23–25]. Therefore, HOPX may be a key factor in cancer cell growth. However, the expression and clinical significance of HOPX in SKCM are still unclear and deserve further study.

In this study, bioinformatics analysis, in vitro experiments, and sequencing validation were used to explore the role of HOPX in cutaneous melanoma for investigation. TCGA, GEO, and HPA databases showed that HOPX was lowly expressed in SKCM and correlated with the diagnosis and prognosis of SKCM patients. To verify the correlation between HOPX and immunity, GO/KEGG analysis, GSVA analysis, immune scoring and single cell sequencing analysis were performed, which showed that HOPX was closely associated with immune infiltration and was most closely related to T cells. In addition, in vitro experiments using human malignant cutaneous melanoma cells showed that HOPX inhibited proliferation, migration, and invasion of A375 cells, promoted apoptosis and S-phase arrest, and may enhance the sensitivity of clinical chemotherapeutic agents. Transcriptome sequencing further analysed the interaction of HOPX with related genes to reveal its possible mechanism of action. The results of this study provide a theoretical basis for the clinical treatment of SKCM.

## Materials and methods

### Cell culture and data mining

The human malignant melanoma cell line A375 was obtained from Boster Biotech (Wuhan, China) and cultured in Dulbecco’s modified Eagle’s medium (DMEM) (Sigma, USA) containing 10% fetal bovine serum (FBS) (Lonsera, UY) and 1% penicillin‒streptomycin (P/S) solution (Beyotime, China) at 37 °C in a humidified incubator (SANYO, Japan) containing 5% CO_2_.

Gene expression and relevant prognostic and clinicopathological data for SKCM patients were collected from The Cancer Genome Atlas (TCGA) and the Gene Expression Omnibus (GEO). The Human Protein Atlas (HPA) database collects clinicopathological and normal sections of skin samples from SKCM patients. Moreover, a full analysis of the data was conducted using the R programming language (version 4.0.3) and R Bioconductor.

### Vector construction and transfection

Using the pcDNA3.1(+) vector and the HindIII and NheI sites, HOPX was amplified and cloned into pcDNA3.1-HOPX. According to the manufacturer’s instructions, Lipofectamine 2000 (Invitrogen, USA) was used to transfect cells with pcDNA3.1(+) (NC) and pcDNA3.1-HOPX (OE).

### RNA extraction, cDNA synthesis and quantitative real-time PCR (RT‒qPCR)

After TRNzol Universal Reagent (TIANGEN, China) was used to prepare cDNA from 2 µg of RNA from cells, the FastKing cDNA Reverse Transcription Kit (TIANGEN, China) was used to prepare cDNA. In addition, the FastKing One Step RT‒qPCR Kit (SYBR) (TIANGEN, China) and an Eppendorf Quantitative PCR Instrument (Eppendorf, China) were used for all RT-qPCRs.

### Cell viability assays

A375 cells were transfected with pcDNA3.1-HOPX for 24 h and cultured in 96-well plates (5000 cell/well) for 0, 24, 48, 72 and 96 h. Then, 10 µL of CCK-8 reagent (MCE, USA) was added to 100 µL of medium per well, and the optical density (OD) value at 450 nm was detected with a microplate reader (TECAN, AUT) after 1.5 h of incubation.

### Wound healing assays

A375 cells were transfected with pcDNA3.1-HOPX for 24 h, the cells were cultured in 6-well plates until confluent, and then excess cells were washed away with PBS; next, a 200 µl pipette tip was used to produce scratches on the cell monolayer. The cells were cultured in serum-free medium, and the wound sites were imaged at 0, 24, and 48 h. The data analysis was performed with ImageJ 2.3.0 software.

### Transwell migration and invasion assays

The cells were trypsinized into single cells and suspended in serum-free medium (at 5 × 10^5^ cell/mL), and then 100 µL of the cell suspension was added to the upper compartment of the Transwell. Then, 500 µL of DMEM with 10% FBS was added to the lower chamber, and the cells were incubated for 24 h. Then, the upper chamber was washed with PBS, and the cells on the upper chamber were wiped off. After fixation with 4% paraformaldehyde, the cells were stained with 0.1% crystal violet. The cells were then washed with PBS, and the membrane was placed on a glass slide and observed under an inverted microscope.

The protocol for invasion assays was basically the same as that for migration assays with the addition of Matrigel (ABW, China); the plate with the coated membrane was placed it at 37 °C for 1 h before embedding cells.

### Cell cycle and apoptosis assays

Based on the manufacturer’s protocol, the cell cycle and apoptosis were analyzed by the Cell Cycle and Apoptosis Analysis Kit (Beyotime, China) and flow cytometry (BECKMAN COULTER, USA). Data analysis was performed with Modifit 3.1.0.0 software[26].

### Western blotting

Proteins were extracted using RIPA lysis buffer (Beyotime, China), and protein levels were quantified using the BCA Protein Assay Kit (Beyotime, China) as indicated by the protocol. Each sample was then separated by 15% SDS‒PAGE and transferred onto PVDF membranes (Immobilon-P, Ireland). These blots were then blocked with 5% nonfat milk at 37 °C for 2 h, after which they were probed overnight with antibodies against human HOPX (1:500, #11419-1-AP, Proteintech) and GAPDH (1:1000, #5174T, CST). Blots were then incubated with appropriate HRP-conjugated secondary antibodies (1:2000, #A0208, Beyotime) for 1 h at 37 °C, after which protein levels were quantified via an ECL Chemiluminescence Kit (Beyotime, China) using an automatic chemiluminescence/fluorescence image analysis system (Tanon 5200, China).

### RNA separation, cDNA library preparation and Illumina sequencing

After the cells were transfected for 24 h, RNA was extracted using TRIzol Reagent (Life Technologies, USA), and RNA quality was assessed. All RNA extractions yielded high-quality RNA. Reverse transcription was performed to create the final cDNA library according to the protocol of the RNA-Seq sample preparation kit (Illumina, USA). We then performed single-end sequencing (50 nt) on the Illumina Novaseq™ 6000 platform by LC Bio Technology Co., Ltd. (Hangzhou, China) following the protocol recommended by the supplier. All technical steps were performed twice. Sequence results were obtained as FPKM per transcript (fragments per kilobase per million read exons) and deposited in the GEO database (GSE221101).

### Functional enrichment analysis

From the TCGA (https://portal.gdc.com), we downloaded RNA sequencing expression (level 3) profiles and clinical information related to SKCM patients. According to the expression level of the *HOPX* gene in the TCGA-SKCM dataset, the patients were divided into two groups: high (n = 236) and low (n = 235), and 455 up-regulated genes and 1 down-regulated gene were obtained. The R package Limma was used to study the differentially expressed mRNAs. Additionally, adjusted *p* < 0.05 and log2 (fold change) > 1 or <-1 were defined as the thresholds for the differential expression of mRNAs. We analyzed the Gene Ontology (GO) function of the underlying mRNAs and enriched Kyoto Encyclopedia of Genes and Genomes (KEGG) pathway by using the ClusterProfiler package in R. In addition, *HOPX* expression in GEO datasets (GSE15605, GSE46517, GSE114445 and GSE100050) was analyzed, and volcano plots were generated using the ggplot package in R.

Sequencing data for the internal cohort (GSE221101) were analyzed with essentially the same protocol as above using the R language (with the same packages mentioned below).

### Gene set variation analysis (GSVA)

We obtained the immune gene list from the Gene Set Enrichment Analysis (GSEA) (http://www.gsea-msigdb.org/) website. We calculated each SKCM sample’s functional enrichment score using default parameters in R. With the pheatmap package in R, we mapped the enrichment results on a heatmap. Pearson correlation was used to determine the correlation between *HOPX* expression and immune responses.

### Immunoscore and correlation analysis

Level 3 RNA sequencing expression profiles and corresponding clinical information for SKCM were downloaded from the TCGA dataset. To assess the reliability of the immune score evaluation, we used immuneeconv. It is an R software package that integrates the six latest algorithms, TIMER, xCell, MCP-counter, CIBERSORT, EPIC and quanTIseq. All the above analysis methods and R package were implemented with R version 4.0.3 and the software packages ggplot2 and pheatmap.

### Analysis of single-cell clusters

The GSE72056 dataset was obtained from the Gene Expression Omnibus (GEO) database (https://www.ncbi.nlm.nih.gov/geo/query/acc.cgi?acc=GSE72056) and processed using the Seurat package in R. Genes expressed in more than three cells were considered expressed, and each cell had to express 200 genes to be included. The FindVariableFeatures function was used to identify the most variable genes from raw UMI counts. The variable genes were used in principal component analysis (PCA). With a resolution of 0.3, the function FindClusters revealed shared nearest neighbors based on PCA using the first 20 principal components. Two-dimensional representations of the cell states were obtained using uniform manifold approximation and projection (UMAP) dimensional reduction analysis. Based on the CellMarker database (http://xteam.xbio.top/CellMarker/) and the literature, the significant genes were used to assign cluster identity to the cell types.

### Analysis of cell stemness

Level 3 RNA sequencing expression profiles and corresponding clinical information for SKCM patients were downloaded from the TCGA dataset, and the OCLR algorithm was used to calculate the mRNAsi. The mRNA gene expression profile included information on 11,774 genes. We used the same Spearman correlation (RNA expression data). The minimum value was subtracted, and the result was divided by the maximum maps of the stemness index to the range [0,1].

### Correlation analysis of RNA expression and IC50

We downloaded level 3 RNA sequencing expression profiles and clinical information related to SKCM from the TCGA dataset. Using the Genomics of Drug Sensitivity in Cancer (GDSC) database (https://www.cancerrxgene.org/), the chemotherapeutic response was predicted for each sample using the pRRophetic package in R. Estimation of the half-maximal inhibitory concentrations (IC50s) was performed by using ridge regression with all parameters set to their default values[27].

### Construction of the ROC, KM and nomogram

Based on the TCGA-SKCM database,469 RNA-sequencing samples were obtained. ROC curves were analysed using the pROC package and visualised by ggplot2 in R. KM curves were statistically analysed using the survival package and the survminer package was used for visualisation. Furthermore, we used clinical TNM staging, radiotherapy and HOPX levels to construct a nomogram to predict 1-, 5-, and 10-year survival rates for SKCM patients. This nomogram was built using the “rms” and “survival” R packages[28].

### Data collection

Based on the GTEx database and TCGA database, SKCM-related data samples were obtained (in TPM format), including 813 normal samples, 469 tumor samples, and unpaired analysis was performed. HPA database to obtain skin tissue sections related to clinical patients. These include Normal Patient-1 (Male, age 51, Patient id: 3298), Normal Patient-2 (Female, age 66, Patient id: 4786), Tumor Patient-1 (Male, age 62, Patient id: 4809) and Tumor Patient-2 (Male, age 62, Patient id: 4809).

### Statistical analyses

All statistical analyses were performed using R (version 4.0.3) software. Kaplan‒Meier survival curves were constructed and compared with log-rank tests. Spearman correlation analysis was used to examine correlations among variables without normal distributions. *p* < 0.05 was considered to indicate statistical significance (ns, p ≥ 0.05, *p < 0.05, **p < 0.01, ***p < 0.001, ****p < 0.0001).

## Results

### Low HOPX expression indicates a poor prognosis in SKCM and HOPX is a good diagnostic marker

**Fig. 1:**
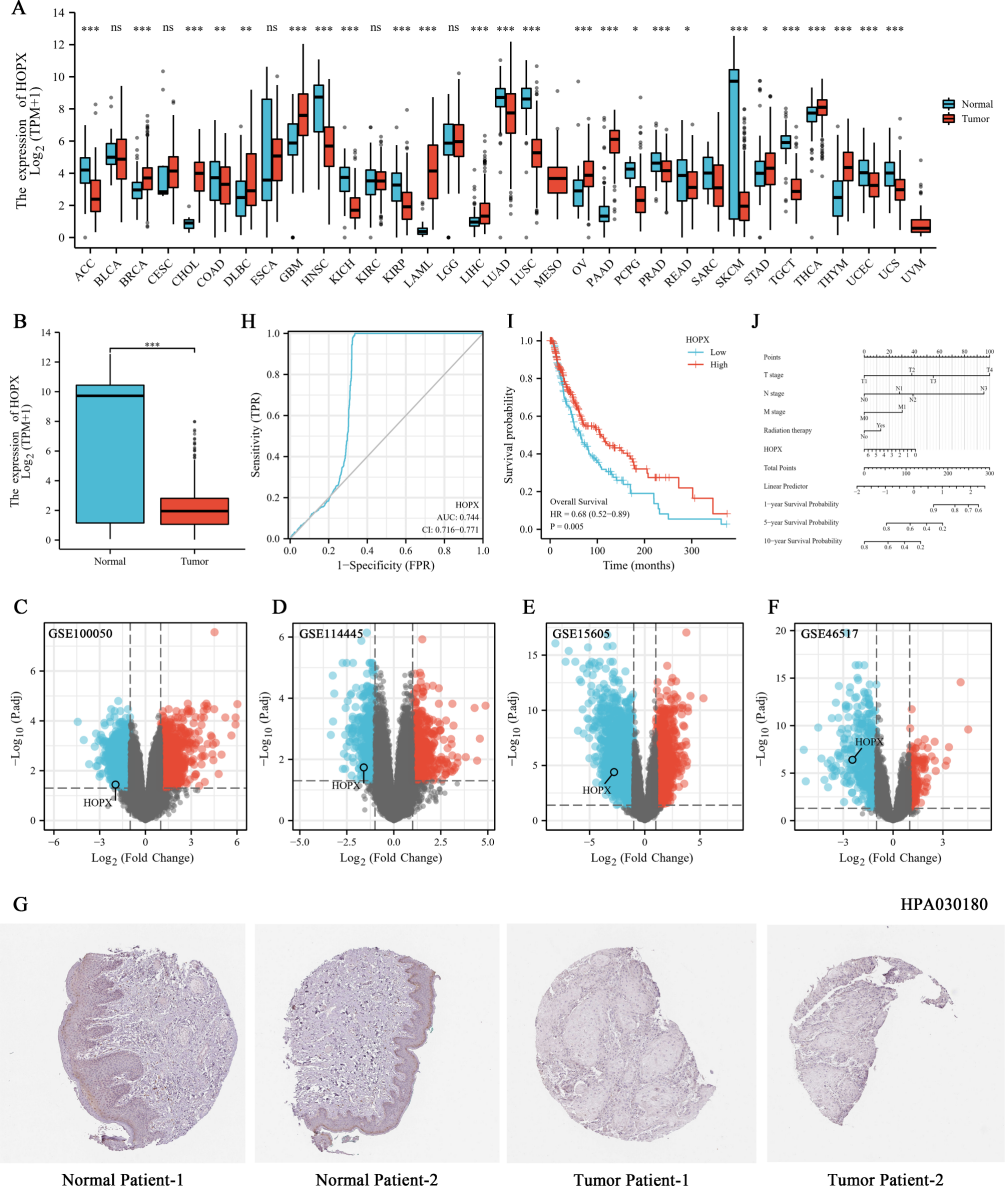
HOPX has diagnostic and prognostic value in SKCM patients. (**A**) Expression of HOPX in paracancer and tumor tissues in TCGA (The Cancer Genome Atlas) database. (**B**) HOPX is expressed at low levels in SKCM patients according to the TCGA database. (**C**-**F**) The GEO (Gene Expression Omnibus) database verified that HOPX is expressed at low levels in SKCM patients. (**G**) Expression of HOPX in skin sections of normal controls and melanoma patients (HPA030180). (**H**) The ROC (receiver-operating characteristic) curve showed the high-expression specificity of HOPX in SKCM patients in TCGA. AUC, area under the curve. (**I**) KM (Kaplan‒Meier) curve showed the better overall survival in SKCM patients with high HOPX expression in TCGA. (**J**) Nomogram predicting 1-, 5-, and 10-year survival rates for SKCM patients based on HOPX expression

The TCGA database was used to analyze the expression of *HOPX* in various normal and tumor tissues. The results showed that *HOPX* expression was significantly upregulated in 25, including ACC (Adrenocortical carcinoma) (*p* < 0.001) and BRCA (Breast invasive carcinoma) (*p* < 0.001) (Fig. 1A), while HOPX expression was much lower in the SKCM tumor group compared to the normal group (*p* < 0.001) (Fig. 1B). The Volcano map results based on the GEO database also showed low expression of HOPX in SKCM patients (Fig. 1C-F). This was also confirmed with clinicopathological data from the HPA database (Fig. 1G). In addition, data analysis revealed no significant differences in HOPX expression levels between age groups among SKCM patient, but expression levels were higher in females than in males (Figure S1A, B). This may mean that men are more likely to develop SKCM and that it may occur at any age, although more clinical data are needed to demonstrate this. Then, we further analyzed the diagnostic and prognostic value of HOPX in SKCM. The receiver operating characteristic (ROC) curve had an AUC of 0.744 (Fig. 1H), indicating the excellent diagnostic value of HOPX in SKCM patients. Moreover, the Kaplan‒Meier (KM) curve indicated that SKCM patients with high HOPX expression had better survival rates (*p* = 0.005) (Fig. 1I). and the nomogram predicted the 1-year, 5-year, and 10-year survival rates for SKCM patients (Fig. 1J). This implies the potential value of HOPX in the diagnosis and prognosis evaluation of SKCM patients.

### *HOPX* is involved in the process of SKCM and may be related to immunity

**Fig. 2:**
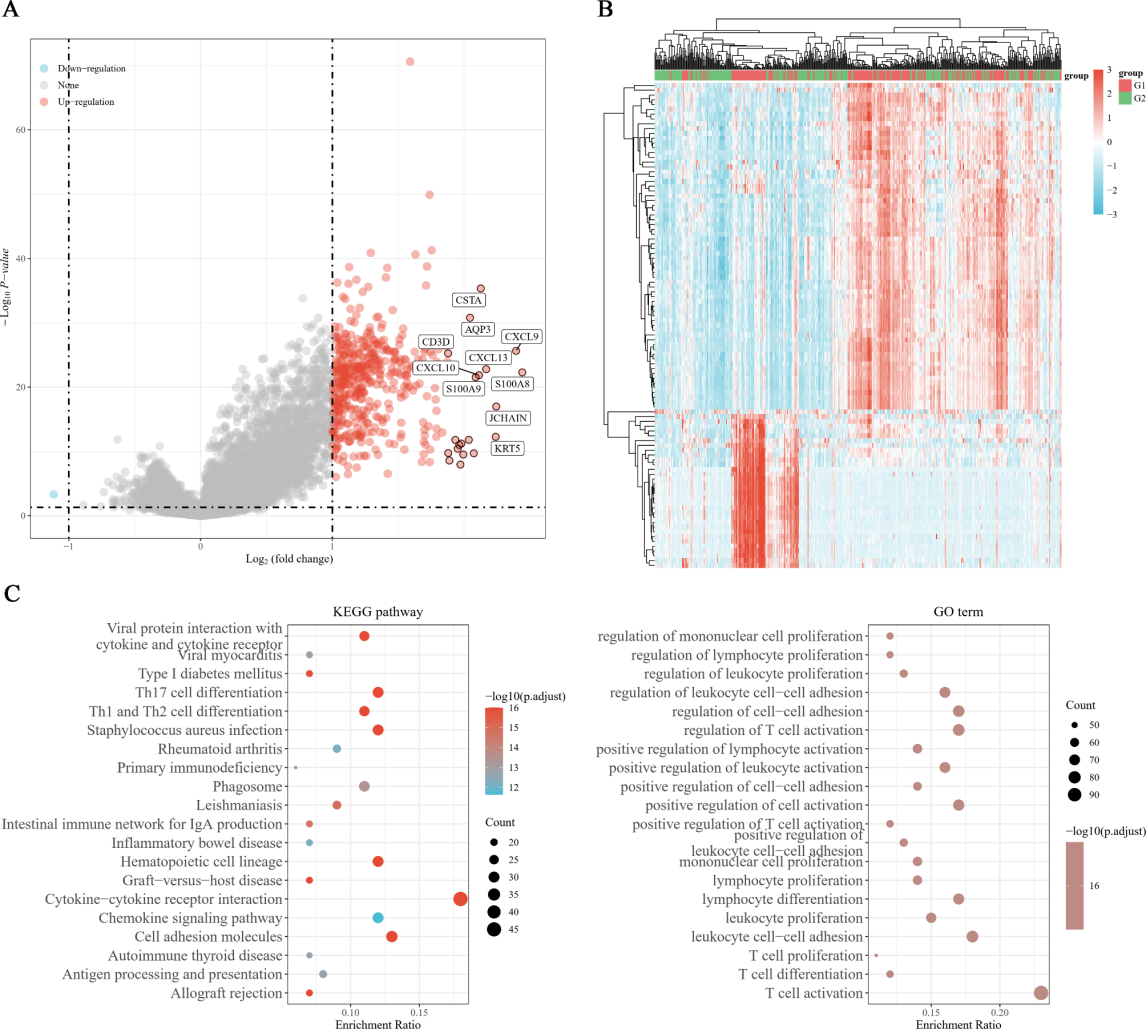
GO and KEGG analysis of HOPX between the G1 subtype and G2 subtype. (**A**) Volcano plot of differentially expressed genes. Red dots indicate upregulated genes; blue dots indicate downregulated genes; grey dots indicate not significant. (**B**) Heatmap showing the differentially expressed genes, different colors represent the trend of gene expression. The top 50 up-regulated genes and top 50 down-regulated genes were showed in this figure. (**C**) KEGG and GO enrichment analysis. Colors represent the significance of differential enrichment, the size of the circles represents the number of genes, the larger the circle, the greater the number of genes. (|Log_2_FC|>1, adjusted P < 0.05)

HOPX expression was investigated in the TCGA database using the LIMMA R package to explore its potential biological function in SKCM. It generated 456 genes; 455 genes were upregulated, and 1 gene was downregulated (|logFC|>1, adjusted *p* < 0.05) (Fig. 2A, B). In the GO and KEGG analyses, HOPX functions were primarily related to cytokine‒cytokine receptor interaction, cell adhesion molecules, leukocyte cell‒cell adhesion, T-cell activation and Th17 cell differentiation (Fig. 2C). Based on these results, we found that HOPX is involved in immune responses in the regulatory role of SKCM.

### *HOPX* inhibited the proliferation, migration and invasion of SKCM cells and promoted cell apoptosis and S-phase arrest

**Fig. 3:**
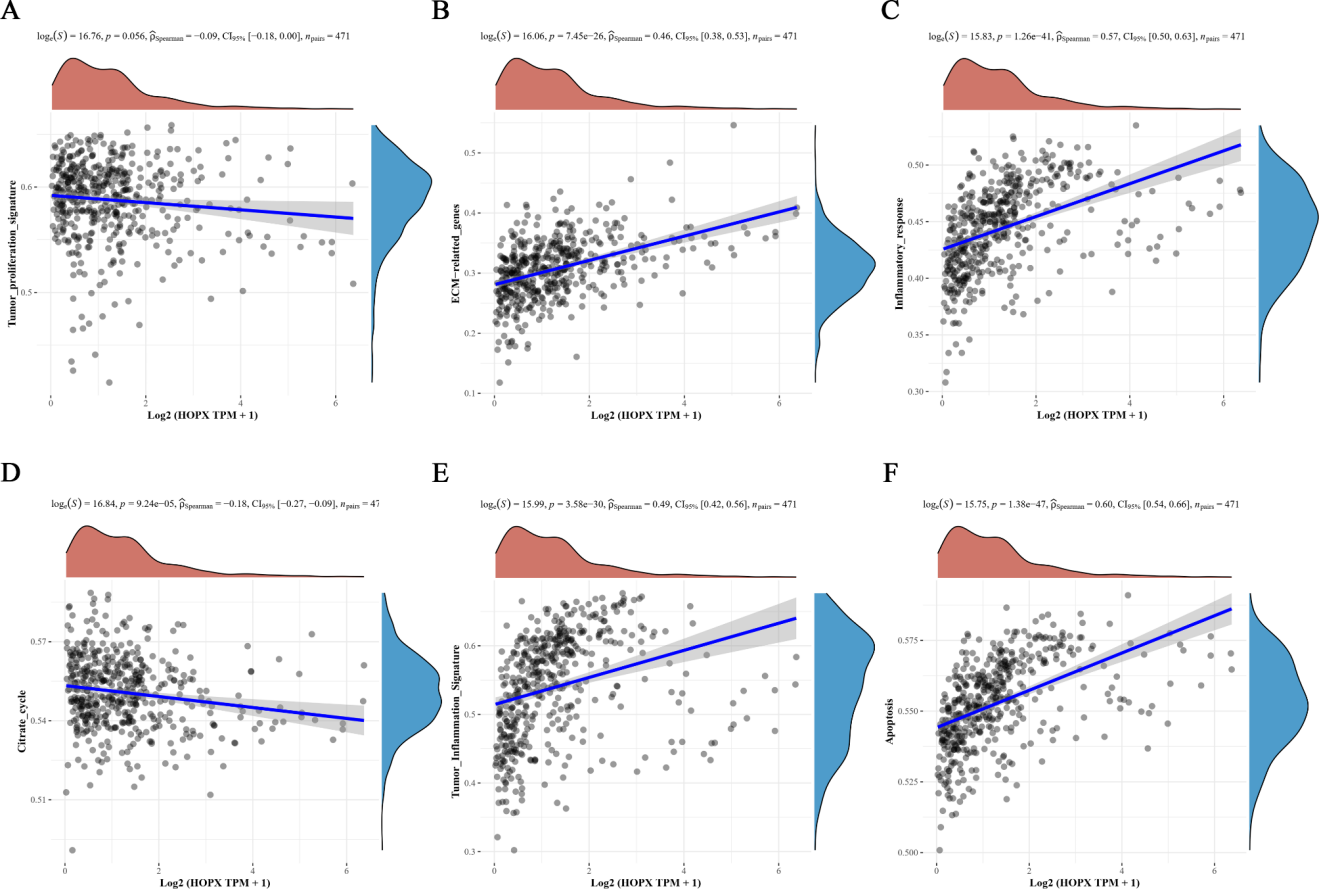
Evaluation of the relationship between HOPX and tumor progression. (**A**) HOPX was negatively correlated with tumor proliferation and the citrate cycle (**D**). (**B**) HOPX was positively correlated with ECM-related genes, inflammatory response (**C**), tumor inflammation signature (**E**) and apoptosis (**F**)

We performed an analysis on the phenotypes associated with HOPX expression in SKCM cell lines, and the correlation analysis showed that HOPX expression was negatively correlated with characteristics such as tumor proliferation and positively correlated with inflammation and apoptosis in SKCM cells (Fig. 3A-F). Therefore, we used the human malignant melanoma cell line A375 for experimental validation.

**Fig. 4:**
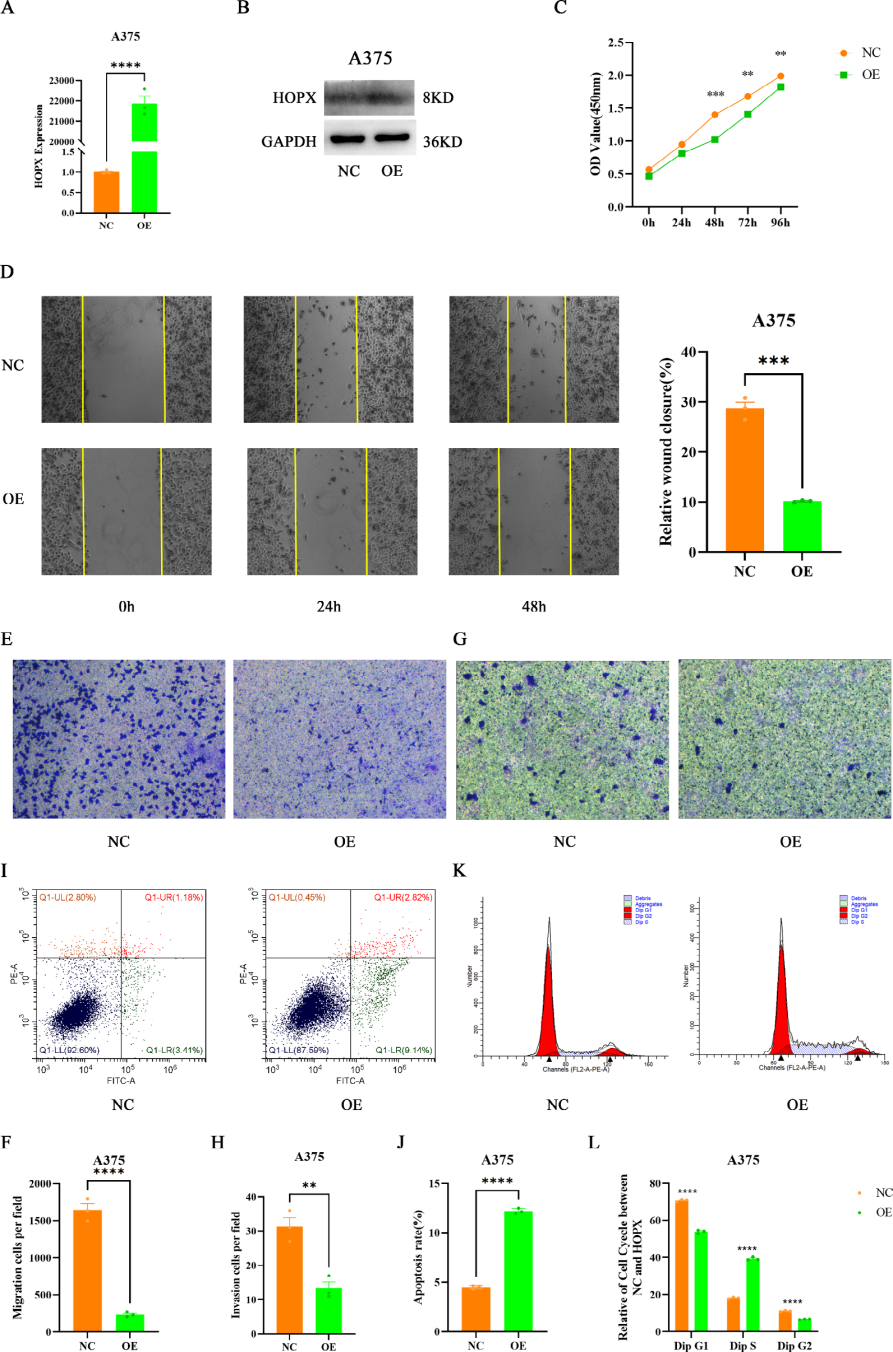
HOPX inhibits proliferation, migration, and invasion and induces apoptosis and cycle arrest in SKCM cell lines. (**A**) The overexpression of HOPX in A375 cells was confirmed by RT‒qPCR and Western blotting (**B**). (**C**) A CCK-8 assay was used to detect the proliferation of A375 cells. (**D**) A wound healing assay was used to detect the migration of A375 cells. (**E**-**H**) Transwell assays were used to detect the migration/invasion of A375 cells. (**I**-**J**) The apoptosis and cell cycle (**K**-**L**) of A375 cells were detected by flow cytometry

First, HOPX was overexpressed in A375 cells, and overexpression efficiency was verified by RT‒qPCR and Western blotting (Fig. 4A, B). The CCK-8 assay showed that HOPX overexpression significantly decreased cell viability, and the effect was most significant at 48 h (Fig. 4C). In addition, wound healing and transwell migration assays showed that HOPX overexpression significantly inhibited cell migration and metastasis (Fig. 4D-F). To further determine whether HOPX affects the invasion of SKCM cells, a transwell invasion assay was performed with A375 cells. The results showed that overexpression of HOPX significantly inhibited the invasion of SKCM cells (Fig. 4G, H). These results suggest that HOPX can reduce cell viability and inhibit cell proliferation, migration and invasion. The role of HOPX in the regulation of apoptosis and the cell cycle was further studied. As expected, overexpressing HOPX potentiated apoptosis (Fig. 4I, J) and cell cycle S-phase arrest (Fig. 4K, L) in A375 cells.

**Fig. 5:**
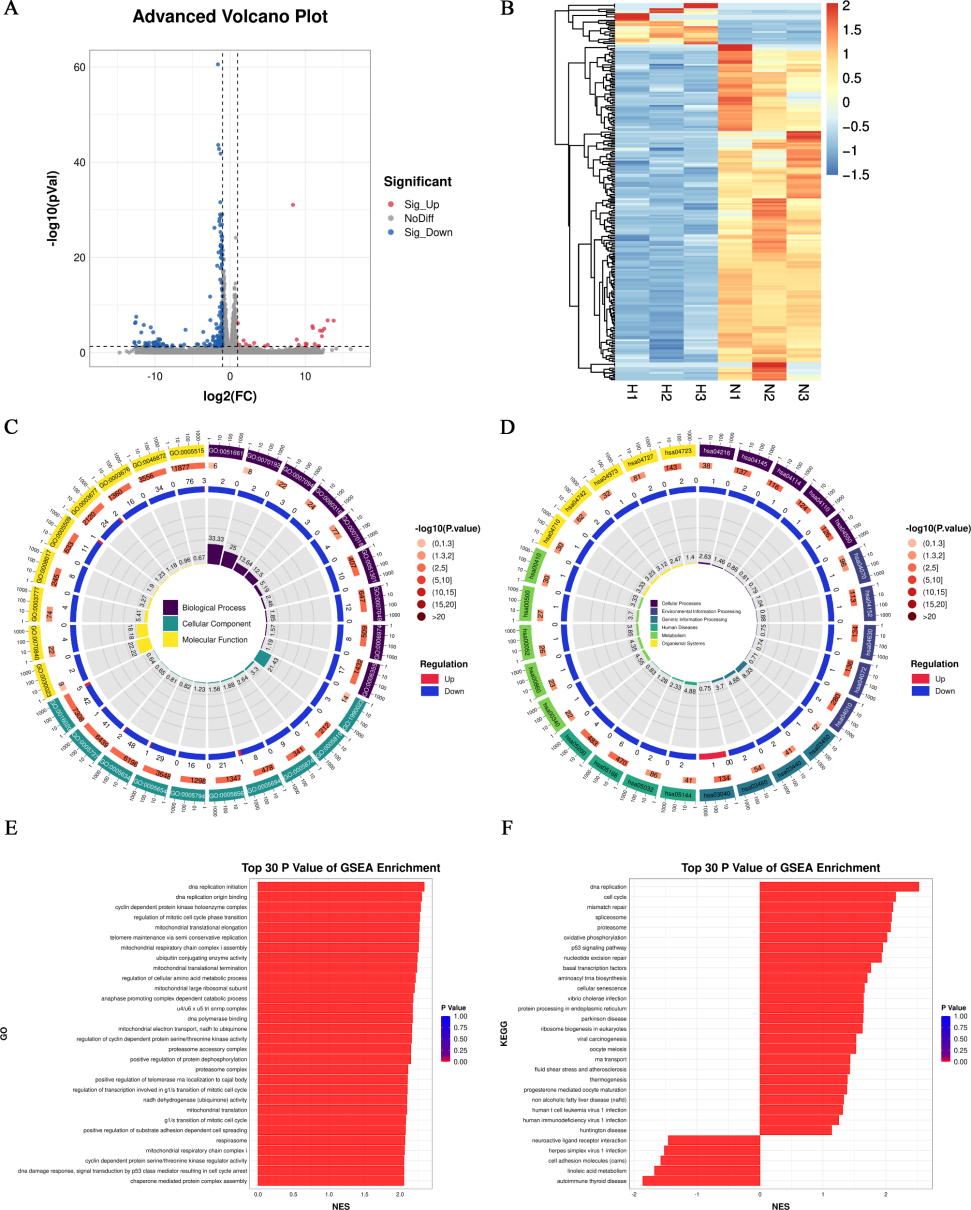
Transcriptome sequencing analysis of HOPX in the SKCM cell line A375. (**A**) Volcano plots were drawn for all genes analyzed for differential expression (|Log_2_FC| >1& adjusted P < 0.05). (**B**) A heatmap was drawn with samples as horizontal coordinates and differentially expressed genes as vertical coordinates. (**C**-**D**) Construction of the GO/KEGG enrichment circle diagram. The first circle (from outside to inside) shows the most GO entry/KEGG pathway (minimum P value), and the number outside the circle indicates the number of enriched genes, with different colors indicating different classifications; the second circle represents the number of genes annotated to the GO entry/KEGG pathway, and the third circle shows the statistics of the number of differentially upregulated and downregulated genes in the GO/KEGG pathway; the fourth circle indicates the percentage of enrichment factors. (**E**-**F**) Construction of GSEA-GO/KEGG enrichment bar graph. The top 30 gene sets with the smallest P and FDR values from GSEA-GO/KEGG analysis are plotted, the horizontal coordinates are the NES values of the gene sets, and the colors represent the P values

Subsequently, we extracted RNA from A375 cells with successful HOPX overexpression for transcriptome sequencing. We compared and counted the differentially expressed genes between the control (N) and overexpression groups (H); there were 25 upregulated genes and 207 downregulated genes (Fig. 5A). According to the similarity of the gene expression profiles of the samples, the genes were clustered and analyzed to construct a heatmap (Fig. 5B). GO, KEGG and GSEA analyses based on sequencing data (GSE221101) showed that the sequencing analysis results were similar to those of the TCGA database analysis results (Fig. 5C-F), suggesting that HOPX is associated with DNA replication, cell cycle, cell division, cytoskeleton, pathways in cancer, regulation of transcription, DNA template and so on. In conclusion, the above results corroborate our experimental findings.

### *HOPX* expression positively correlates with T-cell-mediated immune responses and can serve as an immune checkpoint

Cancer cells death mediated by the immune system involves lymphocyte activation and the release of chemokines and cytokines, and T cells can be recruited to optimize tumor immunotherapy strategies[29].

**Fig. 6:**
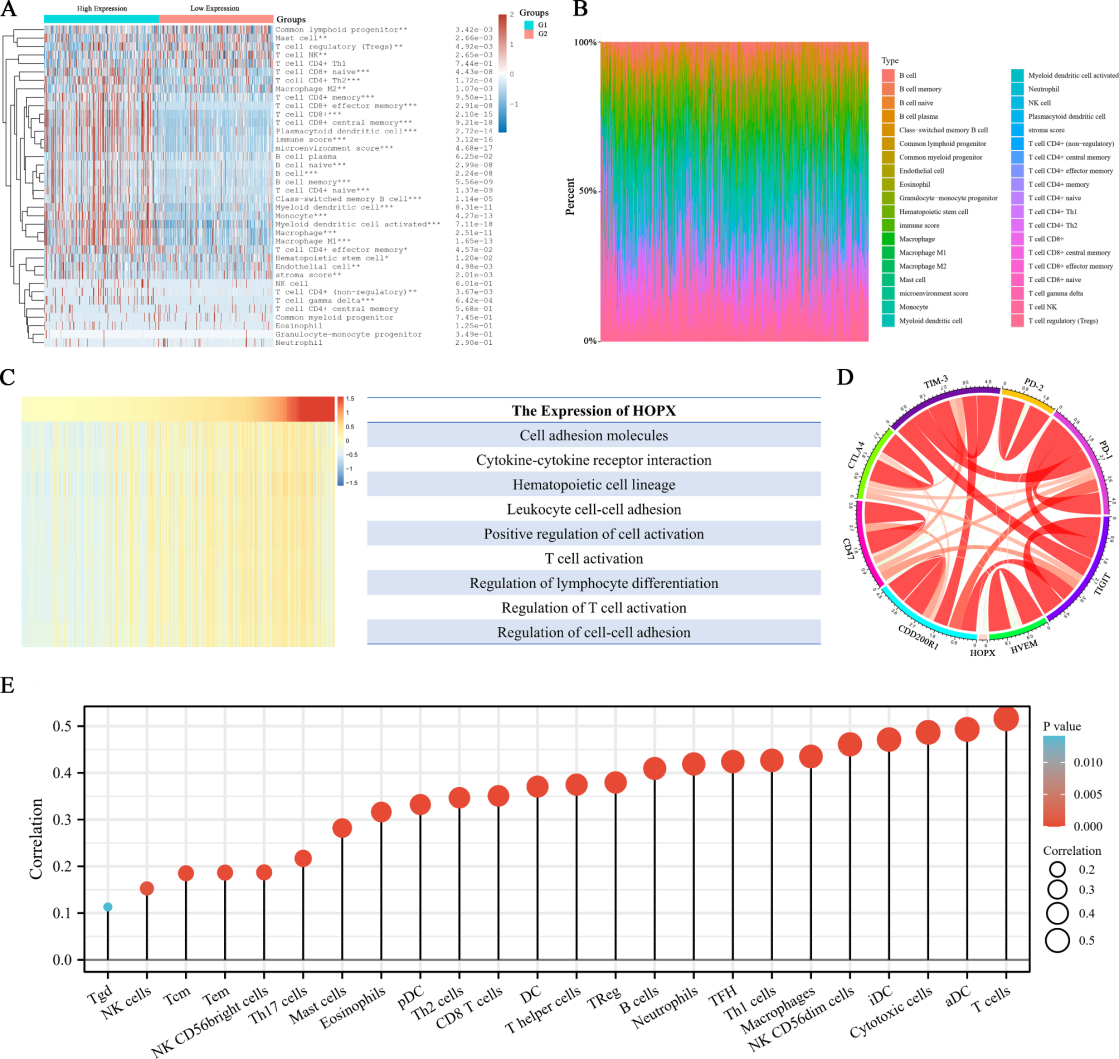
Correlation analysis between HOPX and immune function. (**A**) HOPX immunoscore heatmap of immune cell scores with high and low expression in SKCM tissue and (**B**) percentage abundance of infiltrating immune cells for each sample. (**C**) Heatmap of HOPX and immune function enrichment scores. The heatmap showed the expression of HOPX and the enrichment scores of immune functions of each patient in TCGA databases. (**D**) Pearson correlation between HOPX and immune checkpoints. The width of the band represented the R-value. The color of the band represented the P‐value. The correlation was tested by Pearson correlation analysis. (**E**) Analysis of the correlation between HOPX and immune cells. The size of the circle represents the correlation, with the larger the circle the higher the correlation

Therefore, we investigated the relationship between HOPX expression and immune cells and related cytokines. Level 3 RNA sequencing expression profiles and corresponding clinical information for SKCM patients were downloaded from the TCGA dataset. We used the immuneeconv package to perform immune scoring and Spearman correlation analysis. The results showed that HOPX was involved in the immune response of SKCM, which involved T cells, B cells, macrophages, NK cells, etc. (Fig. 6A, B). The expression of HOPX was positively correlated with the infiltration levels of B cells, T cells (CD4+/8+), neutrophils, macrophages and myeloid dendritic cells (Figure S2A). In contrast, the cell stemness analysis showed that HOPX expression was negatively correlated with the degree of cancer stem cells (CSCs), with significantly higher mRNAsi scores in the SKCM group than in the normal group and lower scores in the high HOPX expression group than in the low expression group (Figure S2B). Gene set variation analysis (GSVA) was used to determine the enrichment fraction of the immune process. The expression of *HOPX* was associated with cell adhesion molecules, cytokine‒cytokine receptor interactions, regulation of T-cell activation, etc. (Fig. 6C). It was also closely related to various immune checkpoints (Fig. 6D). The previous GO/KEGG analysis and immune scoring analyses showed that HOPX expression was associated with the infiltration of multiple immune cell types, while immune cell correlation analysis showed that HOPX expression was most closely related to the infiltration of T cells (Fig. 6E). The above analysis results showed that HOPX expression correlate with immune cell activity and immunotherapeutic response in SKCM patients, and is associated with the highest degree of immune cell, particularly with the highest enrichment of T cells, apart from being a promising immune checkpoint.

### High *HOPX* expression is associated with the infiltration of T cells

The previous data were extensive, but they could not address problems such as intrinsic heterogeneity, so we applied a single-cell sequencing method with high sensitivity and accuracy.

**Fig. 7:**
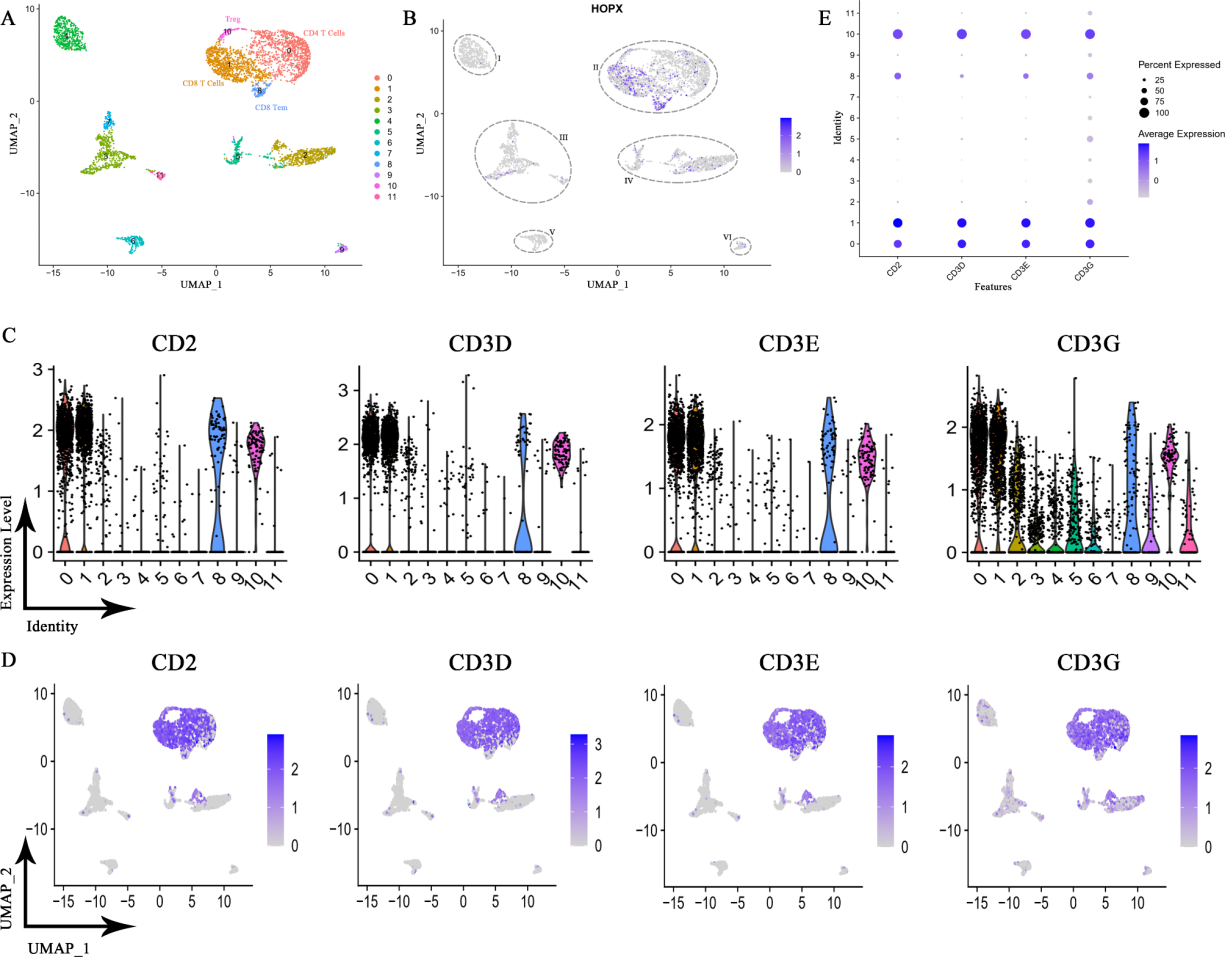
The expression pattern of HOPX in lymphocytes in SKCM. (**A**) Single-cell sequencing analysis demonstrating the cellular subtypes of SKCM, based on the GEO (Gene Expression Omnibus, GSE72056) database. (**B**) HOPX is highly expressed in part II with clusters 1 & 8. (**C**-**E**) T cell markers expression levels in various subtypes. Among the subtypes, T cells are mainly enriched in part II.

We analyzed the public dataset of the GEO database using R. UMAP dimensionality reduction analysis was used to obtain a two-dimensional representation of the cell state, and 12 clusters were created (Fig. 7A). We found that *HOPX* was mainly enriched in part II and highly expressed in cluster 1 and cluster 8 (Fig. 7B). Cellular markers[30] such as CD2, CD3D, CD3E and CD3G classified part II as T cells (Fig. 7C-E), which also agreed with the results of Fig. 5E. Furthermore, we classified each cluster of cells in part II, and the results showed that cluster 0 was CD4 + T cells, cluster 1 was CD8 + T cells, cluster 8 was CD8 Tem cells, and cluster 10 was Treg cells[30–33] (Figure S3, 7A). Collectively, the results suggest that HOPX expression may be associated with the regulation of the immune system, particularly the involvement of immune cells such as T cells. Specifically, there is an association between HOPX expression and the activation and depletion of CD8 + T cells.

### *HOPX* expression is associated with clinical sensitivity to anticancer drugs

**Fig. 8:**
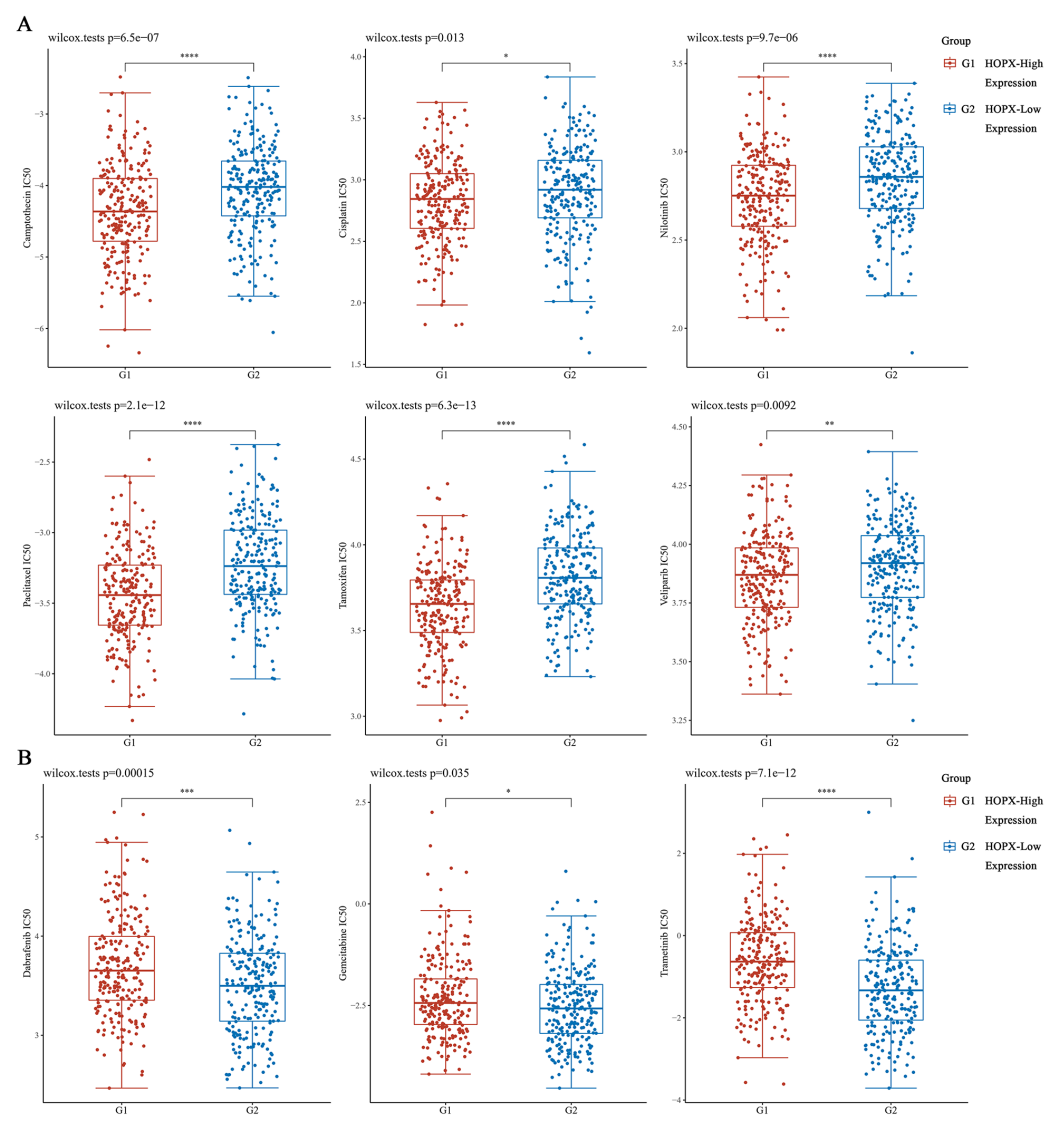
Evaluation of HOPX expression and IC50s for clinical medications. (**A**) High HOPX expression reduces the IC50 values of some clinical drugs and combats drug resistance, (**B**) but the response to other drugs is unaffected in GDSC (genomics of drug sensitivity in cancer) and TCGA (The Cancer Genome Atlas) database

Cancer patients often suffer from drug resistance, which leads to relapse and reduced survival rates. The role of HOPX expression in SKCM drug resistance was therefore studied. Surprisingly, we found a significant correlation between HOPX expression and drug resistance in SKCM. High levels of HOPX were associated with a significant reduction in the IC50 values of several clinical anticancer drugs (including camptothecin, cisplatin, nilotinib, paclitaxel, tamoxifen and veliparib) (Fig. 8A). However, we also found that the IC50 values of dabrafenib, gemcitabine and trametinib were increased in the HOPX high expression group (Fig. 8B). Therefore, HOPX expression levels might be associated with increased sensitivity of cancer cells to clinical drugs and prolonged survival of cancer patients, suggesting its potential as a marker for personalized treatment selection in the clinic.

## Discussion

As a highly malignant tumor, the incidence of melanoma is increasing worldwide and is often closely associated with precursor lesions[34]. The transformation of malignant melanocytes into metastatic melanoma is a complex process caused by the interaction between multiple factors, such as extrinsic, intrinsic, and immune factors; ultraviolet radiation is one of the common major factors involved in the development and progression of melanoma[35]. It has been shown that genes differentially expressed in benign and malignant lesions, as well as those that may have prognostic value, play an important role in tumor progression[36, 37]. In melanoma, altered expression of many genes has been found to be highly correlated with disease progression[38, 39]. Consequently, understanding the relationship between gene expression changes and melanocytes is important for the treatment of tumors as well as the application of precision medicine.

HOPX, also known as NECC1, LAGY, Cameo, and OB1, has an atypical homology domain consisting of 60 amino acids and has three different isoforms, HOPXA, HOPXB, and HOPXC[18, 40]. Initially, HOPX was thought to be associated with cardiac development and the development of the embryonic central nervous system[41, 42]. In this study, based on TCGA-SKCM, GEO, and HPA data and R language analysis, HOPX was found to be expressed at low levels in SKCM tissues relative to normal tissues. To further understand the clinical value of HOPX in SKCM patients, ROC and KM curves showed that HOPX expression levels have better diagnostic value and those with high HOPX expression showed better survival efficiency. Since nomograms have good predictive ability for cancer prognosis[43], one was constructed in this study to predict the survival of SKCM patients at 1, 5 and 10 years, and indeed, patients with high HOPX expression had a better prognosis, which is also the same as the results of H. Ushiku[44] et al. who studied the role of HOPX in pancreatic neuroendocrine tumors.

Furthermore, it has been demonstrated that HOPX plays an important role in the Treg-mediated immune tolerance process and that the number of Tregs is significantly increased in tumor patients and correlates with prognosis[45, 46]. We showed a potential association between HOPX expression and the infiltration of T cells, especially CD8 + T cells, by GO/KEGG analysis, GSVA, immune scoring and single-cell sequencing analysis. T cells, the mainstay in the anti-tumor response, is also considered to be the turning point in immunotherapy. The role of immune checkpoint inhibitors such as CTLA-4 and PD-1 in current clinical treatment is also associated with T cells[47]. Ji P[48] et al. found that LGALS2 was associated with immune cell infiltration and was able to induce macrophage polarization and participate in immunotherapy of breast cancer through CRISPR screening. Modulation of macrophage polarization and thus gene expression levels by regulating macrophages becomes a theoretical approach for the treatment of breast cancer. Similarly, whether the regulation of HOPX expression through the modulation of T cells viability needs to be further explored as a theoretical approach to immunotherapy for SKCM patients.

The HOPX plays a paradoxical role in cancer. On the one hand, HOPX was identified as a tumor suppressor associated with a high degree of promoter methylation leading to epigenetic silencing[49, 50]. On the other hand, it has been shown that HOPX is highly expressed in thyroid cancer and squamous skin cell carcinoma and can promote tumor cell invasion and metastasis[51, 52]. It certainly correlates with the amount of HOPX expression in these solid tumors. Hypermethylation of HOPX-β has been reported to be associated with poor survival in patients with differentiated thyroid cancer, and the methylation level of the HOPX promoter in esophageal squamous carcinoma tissue was inversely correlated with patient survival[53, 54]. In this study, we found that HOPX is under-expressed in SKCM. It is unknown whether its promoter is methylated, which leads to silencing of its expression and thus affects the development of SKCM. In this experiment, we used human malignant melanoma cells as the experimental material and successfully constructed HOPX-overexpressing A375 cells. The results showed that HOPX could inhibit the proliferation, migration and invasion of A375 cells and promote apoptosis and cell cycle arrest. This is also consistent with the results of the correlation analysis in Fig. 3 of this study. A. Ooki[55] et al. found that PAX6 upregulates SOX2 expression and thus promoted stem cell-like transformation of lung cancer cells. This is in turn associated with enhanced expression of key pluripotency factors such as OCT4 and inhibition of differentiation-related factors such as HOPX. Similarly, our results show the potential of HOPX to inhibit tumorigenesis development and also reveal a negative correlation between HOPX expression levels and CSC levels, which is consistent with their findings by A. Ooki. However, it remains to be further explored and verified whether HOPX is associated with the occurrence of promoter methylation in inhibiting A375 cell growth progression to suppress the transformation of cells to stem-like cells.

Melanocytes produce melanin, which is closely linked to oxidative reactions and has antioxidant and pro-oxidant properties[56]. When these cells are stimulated by UV light, heavy metals, and other factors, the cell signaling pathways are overactivated, leading to uncontrolled proliferation, dedifferentiation, and immortalization of specific cell types[57, 58]. When SKCM cells undergo dedifferentiation, it promotes cancer cell metastasis as well as drug resistance and mortality[59, 60]. The combination of immunotherapy and chemotherapy (chemoimmunotherapy) has demonstrated excellent therapeutic effects in the clinical treatment of cancer patients. However, the formation of immunosuppressive niches allows for an increase in drug resistance, which also greatly reduces the therapeutic effect[61, 62]. Therefore, it is essential to reduce drug resistance during tumor treatment, so we performed an analysis of the correlation between clinical drug IC50 and HOPX expression based on the GDSC database. Our results showed a negative correlation between clinical SKCM therapeutic agents and HOPX expression (i.e., clinical agents had lower IC50 values in patients with high HOPX gene expression). However, we also found a positive correlation between the IC50 of clinical drugs such as dabrafenib, gemcitabine, and trametinib and HOPX expression. Therefore, HOPX may have great potential value in clinical treatment, precision medicine application and combating drug resistance.

Overall, we confirmed the practical importance of HOPX in suppressing the development of human malignant melanoma cells, as well as its diagnostic and prognostic value in SKCM patients. Interestingly, HOPX was significantly enriched in SKCM cells and in CD8 + T cells, and HOPX expression and CD8 + T cell infiltration were positively correlated. Furthermore, HOPX plays an integral role in reducing the IC50 of clinical drugs. As a result, HOPX represents a potential therapeutic target in patients with cutaneous melanoma and has the potential to be used as a diagnostic and prognostic factor. To validate these findings, further experiments are needed.

## Conclusion

In summary, our study suggests that HOPX can be used as a diagnostic and prognostic marker in SKCM patients and that it may participate in tumor immunotherapy by modulating the biological activity of various immune cells and attenuating clinical drug resistance in patients. In addition, HOPX may serve as a novel molecular biomarker for skin cutaneous melanoma treatment, which may facilitate the development of novel immunotherapy strategies with high clinical significance in the future.

## Electronic supplementary material

Below is the link to the electronic supplementary material.

Supplementary Material 1

Supplementary Material 2

Supplementary Material 3

## Data Availability

The datasets analyzed for this study can be found on the TCGA-SKCM project (http://www.cancer.gov/tcga), GTEx (https://gtexportal.org/), GEO (https://www.ncbi.nlm.nih.gov/geo/query/acc.cgi?acc=GSE72056/GSE100050/GSE114445/GSE15605/GSE46517), HPA (https://www.proteinatlas.org/) and GDSC (https://www.cancerrxgene.org/) websites. The gene transcriptome sequencing data in this study can be found in the NCBI Gene Expression Omnibus (GEO), record number GSE221101. Original data referenced in the study are included in the article/supplementary materials, and further inquiries can be directed to the corresponding author.

## Abbreviations

AUC: area under the curve
CSCs: cancer stem cells
GDSC: genomics of drug sensitivity in cancer
GEO: gene expression omnibus
GO: gene ontology
GSEA: gene set enrichment analysis
GSVA: gene set variation analysis
GTEx: genotype-tissue expression
HPA: human protein atlas
IC50: half-maximal inhibitory concentration
KEGG: Kyoto encyclopedia of genes and genomes
KM: Kaplan‒Meier
ROC: receiver-operating characteristic
SKCM: skin cutaneous melanoma
TCGA: The Cancer Genome Atlas
