# Supplementary material for: HOPX is a tumor-suppressive biomarker that corresponds to T cell infiltration in skin cutaneous melanoma
Source: Cancer Cell Int. 2023 Jun 21;23:122. doi: 10.1186/s12935-023-02962-2 (PMC10286411; doi:10.1186/s12935-023-02962-2)
Supplement: Supplementary file 1 — Supplementary Material 1 [file 12935_2023_2962_MOESM1_ESM.docx]

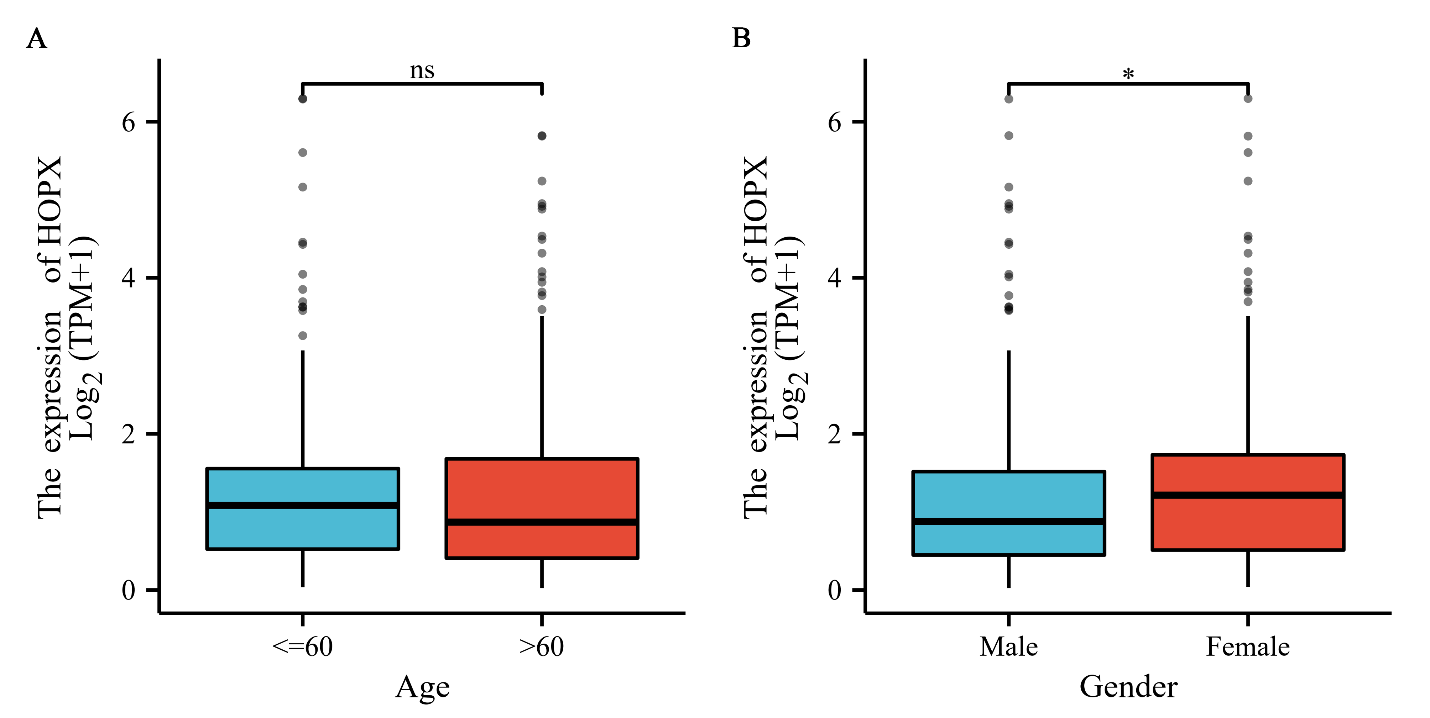


**Figure S1. Expression of HOPX in age and sex groups in the TCGA-SKCM cohort.** (A) HOPX did not differ significantly between the ≤60 and >60 groups. (B) HOPX differed significantly between the male and female groups.
