# Supplementary material for: HOPX is a tumor-suppressive biomarker that corresponds to T cell infiltration in skin cutaneous melanoma
Source: Cancer Cell Int. 2023 Jun 21;23:122. doi: 10.1186/s12935-023-02962-2 (PMC10286411; doi:10.1186/s12935-023-02962-2)
Supplement: Supplementary file 2 — Supplementary Material 2 [file 12935_2023_2962_MOESM2_ESM.docx]

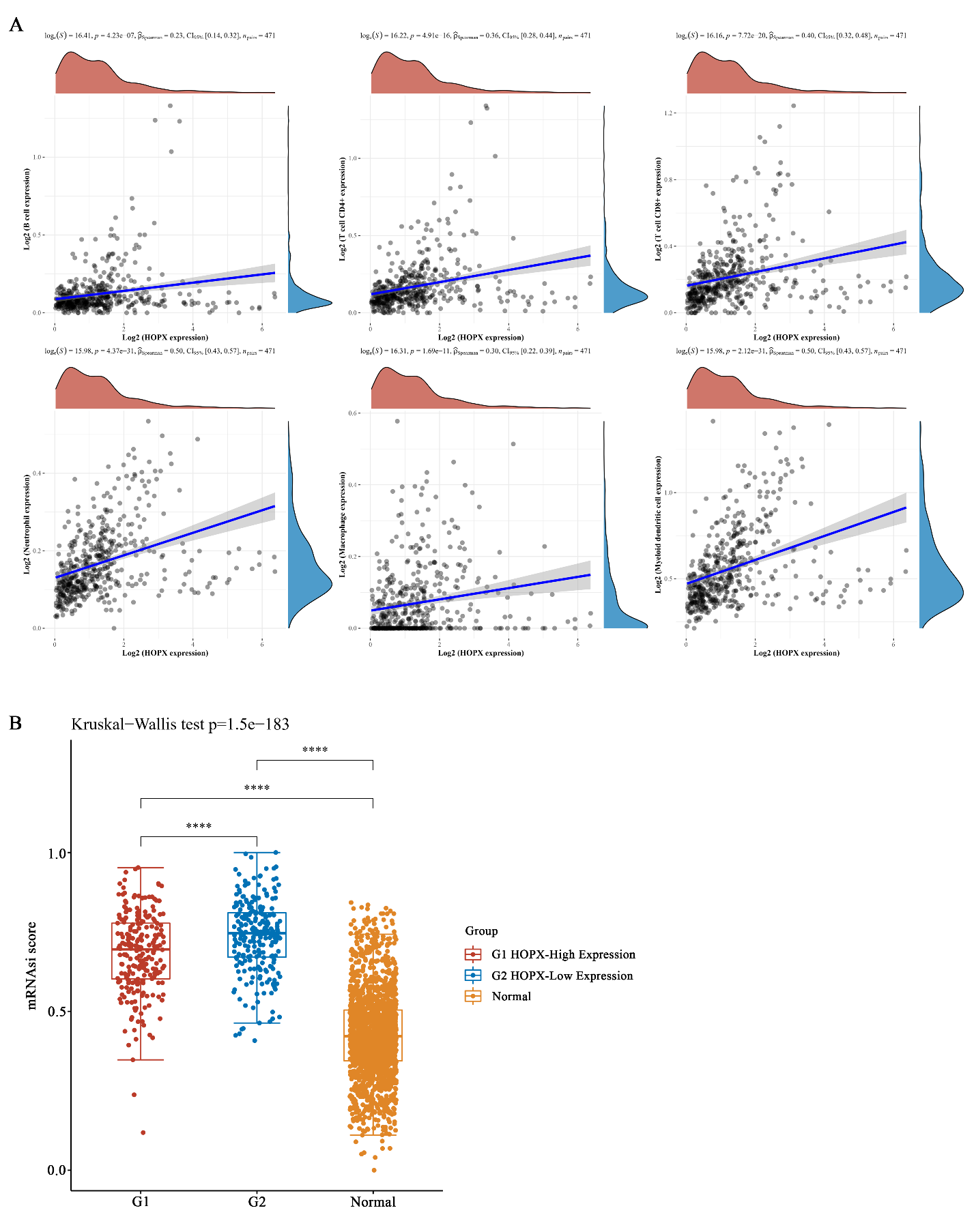


**Figure S2. Assessing the relationship between HOPX and immune cells.** (A) The correlations between HOPX expression and immune score were analyzed with Spearman’s test. (B) mRNAsi scores between high and low HOPX expression groups and normal groups. The higher the expression of HOPX, the lower the degree of CSCs, and the two are negatively correlated
