# Supplementary material for: HOPX is a tumor-suppressive biomarker that corresponds to T cell infiltration in skin cutaneous melanoma
Source: Cancer Cell Int. 2023 Jun 21;23:122. doi: 10.1186/s12935-023-02962-2 (PMC10286411; doi:10.1186/s12935-023-02962-2)
Supplement: Supplementary file 3 — Supplementary Material 3 [file 12935_2023_2962_MOESM3_ESM.docx]

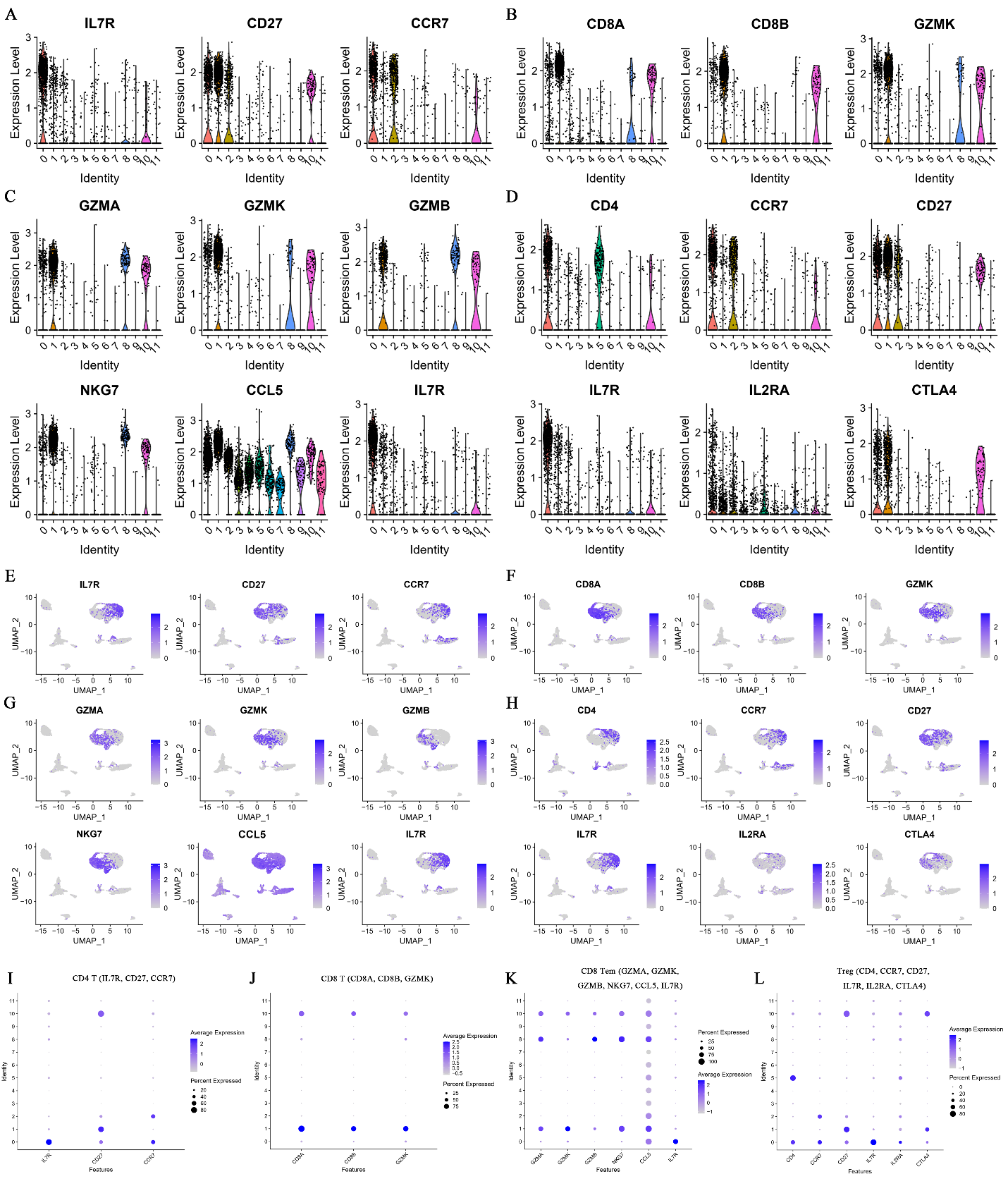


**Figure S3. Validation of HOPX in single-cell sequencing for each cluster biomarker in part II.** (A, E, I) Cluster 0 represents CD4 T cells indicated by 3 molecular markers. (B, F, J) Cluster 1 represents CD8 T cells indicated by 3 molecular markers. (C, G, K) Cluster 8 represents CD8 Tem cells indicated by 6 molecular markers. (D, H, L) Cluster 10 represents Tregs indicated by 6 molecular markers.
